# Editorial: Update on the Endocrinology of Myocardial Aging/Heart Failure

**DOI:** 10.3389/fendo.2020.580948

**Published:** 2020-10-15

**Authors:** Yang Yang, Russel J. Reiter, Shuai Jiang

**Affiliations:** ^1^Faculty of Life Sciences, Northwest University, Xi'an, China; ^2^Department of Cell Systems and Anatomy, UT Health San Antonio, San Antonio, TX, United States; ^3^Department of Aerospace Medicine, The Air Force Military Medical University, Xi'an, China

**Keywords:** myocardial aging, heart failure, metabolism, sepsis, diabetes

Cardiovascular diseases (CVDs), including cardiomyopathy, heart failure, hypertension, and atherosclerosis, have become the leading cause of death worldwide (1). The continuous increase in CVDs is partially due to the increased number of aging populations. Aging, now considered as a core and independent risk factor for the development of CVDs, is characterized by gradually declined physiological function, disturbed repair mechanisms, senescence, and eventually death. In aged and pathological myocardial tissues, maladaptation of cellular metabolism, dysfunction (or senescence) of cardiomyocytes, decrease in angiogenesis, and increase in tissue scarring (fibrosis) are observed (2–4). Senescent cardiomyocytes exhibit the hallmarks of DNA damage, endoplasmic reticulum (ER) stress, mitochondria dysfunction, contractile dysfunction, hypertrophic growth, and senescence-associated secreting phenotype (SASP), all of which finally contribute to cardiac aging, dysfunction, and failure (4). Heart failure is defined as the inability of the heart to supply the peripheral tissues with the required amount of blood and oxygen to meet their metabolic demands, which leads to symptoms like dyspnea or fatigue and signs including elevated jugular venous pressure, tachycardia, or peripheral edema (5). Heart failure is mostly caused by an underlying myocardial disease; however, valve diseases, endocardial or pericardial abnormalities, disorders in the heart rate/rhythm, and aging may also result in cardiac malfunction (6).

The endocrine system is a complex network system that regulates virtually all biological processes, including development, growth, reproduction, metabolism, and responses to stressors (7, 8). Aging leads to significant alterations in the endocrine system, but on the other hand, imbalances in the endocrine system also affect the aging process (7–9). For instance, the secretory patterns of hormones produced by the hypothalamic-pituitary axis as well as the sensitivity to hormones by their target organs are altered in the elderly population. Conversely, imbalances in the production of hormones or alterations in their negative feedback loops have been shown to accelerate the aging process by causing disturbances in metabolism, cardiovascular function, and cognition (7, 8).

The Research Topic covers the themes of sepsis, aging, heart failure, cardiac metabolism, and diabetes. Di et al. found that melatonin significantly increased the survival rate after lipopolysaccharide (LPS)-induced shock. In the sepsis model, melatonin markedly ameliorated myocardial dysfunction by decreasing the release of inflammatory cytokines, activating AMP-activated protein kinase (AMPK) and autophagy, and improving mitochondrial function. Furthermore, they confirmed that AMPK inhibition down-regulated autophagy and abolished the protection of melatonin. Notably, autophagy inhibition by 3-Methyladenine (3-MA) also significantly impaired the protective effects of melatonin, whereas autophagy activation by Rapamycin reversed LPS+Compound C-induced myocardial injury. Besides, *in vitro* studies further confirmed the protection of melatonin against LPS-induced myocardial injury and the mechanisms involving AMPK-mediated autophagy signaling (Di et al.). Bruno et al. investigated 84 patients diagnosed with heart failure with preserved ejection fraction (HFpEF) and revealed a high prevalence of anabolic deficiencies in HFpEF. As indicated in their research, dehydroepiandrosterone-sulfate seems to influence antioxidant levels; insulin-like growth factor-1 deficiency was associated with alterations in parameters of myocardial structure and dysfunction. These data suggest a role of anabolic hormones in the complex pathophysiological mechanisms of HFpEF, which represents the basis for longitudinal studies and investigations on possible benefits of replacement therapy (Bruno et al.).

In addition, Barrientos et al. concluded that testosterone-related metabolic signaling and gene expression might constitute a relevant therapeutic target for preventing or treating age- and gender-related cardiometabolic diseases in men. They also thoroughly discussed how cardiac metabolism is regulated by androgen levels in aging men (Barrientos et al.). Tang et al. first summarized the hallmarks of the senescence of cardiomyocytes. Then, they discussed the metabolic switch within senescent cardiomyocytes and the cellular communications between dysfunctional cardiomyocytes and non-myocytes in local microenvironment. Moreover, they also addressed the function of metabolic regulators within non-myocytes in modulating myocardial microenvironment (Tang et al.). Hu et al. investigated the association of circulating fibroblast growth factor 19 (FGF19) levels with the development of subclinical atherosclerosis (subAS) in patients with type 2 diabetes (T2D) in a 3-year prospective study and found serum FGF19 levels were positively correlated with carotid intima-media thickness (IMT) and iliac IMT in men. In this 3-year follow-up, 25 out of 153 patients developed subAS, and FGF19 levels in men were higher in the subAS group than in the non-subAS group. Alongside with more detailed data, serum FGF19 levels could help to predict the development of atherosclerosis in men with T2D (Hu et al.). Cruz-Topete et al. summarized the positive and negative effects of glucocorticoids on the heart and provided the latest molecular and physiological evidence on how alterations in glucocorticoid signaling lead to changes in cardiac structure and function. They also briefly discussed the effects of other hormone systems such as estrogens and growth hormone/insulin growth factor-1(GH/IGF-1) on the cardiovascular system during aging. They will also reviewed the link between imbalances in glucocorticoid levels and the molecular processes responsible for promoting cardiomyocyte dysfunction in aging. Additionally, they discussed the potential for selectively manipulating glucocorticoid signaling in cardiomyocytes, which may represent an improved therapeutic approach for preventing and treating age-related heart disease (Cruz-Topete et al.).

The Research Topic highlights some of the recent findings of sepsis, aging, heart failure, cardiac metabolism, diabetes. I would like to thank all the authors and reviewers for their contribution and discussion to put together this excellent topic that may inspire further interest in this exciting new field.

## Author Contributions

All authors listed have made a substantial, direct and intellectual contribution to the work, and approved it for publication.

## Conflict of Interest

The authors declare that the research was conducted in the absence of any commercial or financial relationships that could be construed as a potential conflict of interest.

## References

[1] Miranda JJ Barrientos-Gutiérrez T Corvalan C Hyder AA Lazo-Porras M Oni T. Understanding the rise of cardiometabolic diseases in low- and middle-income countries. Nat Med. (2019) 25:1667–79. 10.1038/s41591-019-0644-7 31700182

[2] Picca A Mankowski RT Burman JL Donisi L Kim JS Marzetti E. Mitochondrial quality control mechanisms as molecular targets in cardiac ageing. Nat Rev Cardiol. (2018) 15:543–54. 10.1038/s41569-018-0059-z 30042431 PMC6283278

[3] Roh J Rhee J Chaudhari V Rosenzweig A. The role of exercise in cardiac aging: from physiology to molecular mechanisms. Circ Res. (2016) 118:279–95. 10.1161/circresaha.115.305250 26838314 PMC4914047

[4] Gude NA Broughton KM Firouzi F Sussman MA. Cardiac ageing: extrinsic and intrinsic factors in cellular renewal and senescence. Nat Rev Cardiol. (2018) 15:523–42. 10.1038/s41569-018-0061-5 30054574

[5] Tanai E Frantz S. Pathophysiology of heart failure. Compr Physiol. (2015) 6:187–214. 10.1002/cphy.c140055 26756631

[6] Islam MS. Heart failure: from research to clinical practice. Adv Exp Med Biol. (2018) 1067:1–3. 10.1007/5584_2018_181 29500793

[7] Cai H McNeilly AS Luttrell LM Martin B. Endocrine function in aging. Int J Endocrinol. (2012) 2012:872478. 10.1155/2012/872478 23346110 PMC3546474

[8] van den Beld AW Kaufman JM Zillikens MC Lamberts SWJ Egan JM van der Lely AJ. The physiology of endocrine systems with ageing. Lancet Diabetes Endocrinol. (2018) 6:647–58. 10.1016/s2213-8587(18)30026-3 30017799 PMC6089223

[9] Chiao YA Rabinovitch PS. The aging heart. Cold Spring Harb Perspect Med. (2015) 5:a025148. 10.1101/cshperspect.a025148 26328932 PMC4561390

